# Prevalence of and Factors Associated with Antibiotic Prescriptions in Patients with Acute Lower and Upper Respiratory Tract Infections—A Case-Control Study

**DOI:** 10.3390/antibiotics10040455

**Published:** 2021-04-16

**Authors:** Winfried V. Kern, Karel Kostev

**Affiliations:** 1 Department of Medicine II, Division of Infectious Diseases, University Hospital and Medical Center, 79106 Freiburg, Germany; winfried.kern@uniklinik-freiburg.de; 2 Epidemiology, IQVIA Germany, 60549 Frankfurt am Main, Germany

**Keywords:** antibiotic prescription, primary care, risk factors, phytopharmaceutical, Germany

## Abstract

Background: The goal of the present study was to estimate the prevalence of patient and physician related variables associated with antibiotic prescriptions in patients diagnosed with acute lower and upper respiratory tract infections (ALURTI), treated in general practices (GP) and pediatric practices, in Germany. Methods: The analysis included 1,140,095 adult individuals in 1237 general practices and 309,059 children and adolescents in 236 pediatric practices, from the Disease Analyzer database (IQVIA), who had received at least one diagnosis of an ALURTI between 1 January 2015 and 31 March 2019. We estimated the association between 35 predefined variables and antibiotic prescription using multivariate logistic regression models, separately for general and pediatric practices. The variables included the proportion (as a percentage) of antibiotics or phytopharmaceuticals on all prescriptions per practice, as an indicator of physician prescription preference. Results: The prevalence of antibiotic prescription was higher in patients treated in GP (31.2%) than in pediatric practices (9.1%). In GP, the strongest association with antibiotic prescription was seen in the practice preference for antibiotic use, followed by specific diagnoses (acute bronchitis, sinusitis, pharyngitis, laryngitis, and tracheitis), and higher patient age. In pediatric practices, acute sinusitis and bronchitis were the variables with the strongest association, followed by practice preference for antibiotic prescription. The strongest association with the non-prescription of antibiotics was practice preference for phytopharmaceuticals and the specific diagnosis of a viral infection. Conclusion: This study shows a high prevalence of antibiotic prescribing for patients with ALURTI in a primary care setting, especially in adult patients; physician related factors play an important role that should be addressed in interventions to reduce potentially inappropriate antibiotic prescribing.

## 1. Introduction

Common colds, defined as acute lower and upper respiratory tract infections (ALURTI), are benign, self-limiting syndromes that include a group of diseases caused by several virus families. The severity and type of symptoms vary depending on the person and the pathogen [1]. ALURTI have a high prevalence worldwide. In Germany, the average 1-year prevalence of ALURTI in children and adolescents is about 89% [2]. In adults, ALURTI occur less frequently.

Although physicians know that ALURTI are primarily caused by viruses, antibiotics are prescribed in many cases [3]. However, most patients suffering from ALURTI do not benefit from antibiotic treatment. Inappropriate use of antibiotics, in particular in the ambulatory setting and in veterinary medicine, contributes to an increasing bacterial drug resistance [4,5] and is considered one of the major concerns to governments worldwide [6,7]. There is evidence of increasing environmental resistance that correlates with the use of these drugs and with their industrial-scale production [8]. The decreasing efficacy of antibacterial drugs has come with substantial costs that are expected to increase throughout future decades. One study has predicted that the cumulative global cost of antibiotic resistance might exceed 100 trillion USD [9]. It has been estimated that worldwide at least 700,000 people die annually as a result of resistant infections [9].

Data from Germany revealed that 40–45% of all outpatient antibiotic prescriptions were written by general practitioners [10]. Many of the antibiotic prescriptions were for patients with acute respiratory conditions, including sinusitis, pharyngitis, viral upper respiratory tract infections, bronchitis, and others [11], and a substantial proportion is likely to have been inappropriate. To reduce inappropriate antibiotic prescribing by physicians practicing in outpatient settings, it is important to better understand the variables associated with doctors’ antibiotic prescribing decisions. No data are published yet on the frequency of inappropriate antibiotic use for ALURTI in Germany, and there are no data for factors associated with antibiotic prescriptions in patients with ALURTI in this country.

The goal of the present study was to estimate the prevalence of antibiotic use, and patient and physician related variables associated with antibiotic prescriptions in patients diagnosed with ALURTI, treated in general and pediatric practices in Germany.

## 2. Methods

### 2.1. Data Source

This study was based on data from the IMS Disease Analyzer database (IQVIA), which contains drug prescriptions, diagnoses, and basic medical and demographic data obtained directly, and in an anonymous format from computer systems used in the practices of general practitioners and specialists, including pediatricians [12]. The database covers approximately 3% of all outpatient practices in Germany. Diagnoses (according to International Classification of Diseases, 10th revision (ICD-10)), prescriptions (according to Anatomical Therapeutic Chemical (ATC) Classification system), and the quality of reported data, are monitored by IQVIA. The sampling method for the Disease Analyzer database is based on summary statistics from all doctors in Germany, published yearly by the German Medical Association. IQVIA uses these statistics to determine the panel design according to the following strata: specialist group, German federal state, community size category, and age of physician. The representativeness of the Disease Analyzer database has been previously verified [12].

### 2.2. Study Population

**Figure 1:**
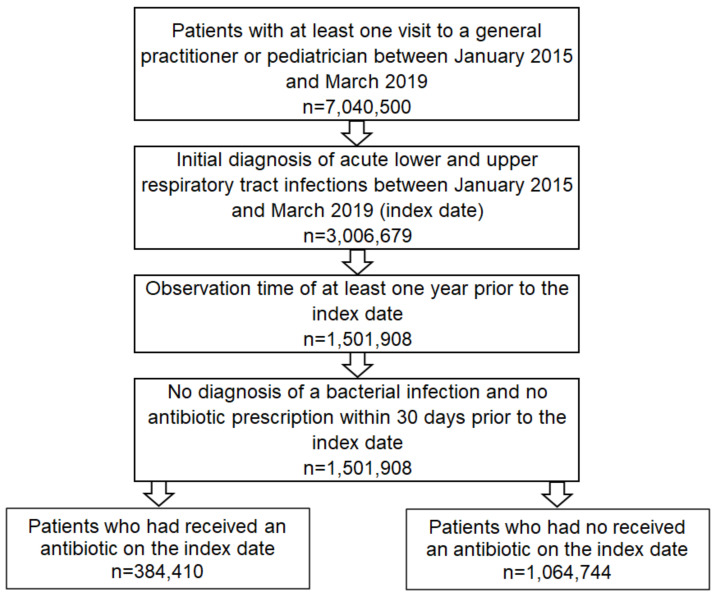
Selection of study patients from IMS Disease Analyzer database.

The analysis included patients who had received at least one diagnosis of ALURTI in 1237 general or 236 pediatric practices between January 1, 2015 and March 31, 2019. These diagnoses included: acute nasopharyngitis (J00), acute sinusitis (J01), acute pharyngitis (J02, excl. J02.0), acute laryngitis and tracheitis (J04), acute upper respiratory infections of multiple and unspecified sites (J06), acute bronchitis (J20, excl. J20.0, J20.1, and J20.2), unspecified acute lower respiratory infection (J22), bronchitis not specified as acute or chronic (J40), cough (R05), and viral infection of an unspecified site (B34).The first ALURTI diagnosis documented during this period was defined as the index date. Only patients who had been observed for a period of at least 12 months prior to the index date were included. Patients with a prescription for antibiotics (ATC code: J01) in the 30 days prior to the index date, and patients diagnosed with a bacterial infection (A38, J02.0, J03.0, J13-J15, J16.0, J20.0, J20.1, J20.2, B95, B96) at or in the 30 days prior to the index date, were excluded from the study (Figure 1).

### 2.3. Study Outcomes and Coviariables

The main outcome of the study was the proportion of patients with ALURTI who received an antibiotic prescription on the day of diagnosis. Additionally, we estimated the association between predefined variables and antibiotic prescription. Variables used included age, sex, health insurance coverage, region (West versus East Germany), ALURTI diagnoses by ICD-10 codes, chronic co-diagnoses documented within 12 months prior to or on the index date (diabetes (ICD-10: E10-14)), ischemic heart disease/heart failure (ICD-10: E20-25, I50), renal failure (ICD-10: E10.2, E11.2, E14.2, N18, N19), cancer (ICD-10: C00-C98), chronic obstructive lung disease (COPD) (ICD-10: J44), asthma (ICD-10: J45)), diagnosis of a bacterial infection within 365–31 days prior to the index date, and the total number of patients per practice and quarter. Finally, two variables were used to consider the extent of the antibiotic and phytopharmaceutical preference of each practice: average proportion of antibiotic prescription on all prescriptions per practice, and average proportion of phytopharmaceutical prescription on all prescriptions per practice within twelve months prior to the index date.

### 2.4. Statistical Analyses

The prevalence of antibiotic prescription was calculated as the number of patients with at least one antibiotic prescription on the index date by the number of all patents with ALURTI.

Patients were grouped by those who had received and those who had not received an antibiotic on the index date. Differences in the sample characteristics between those with and those without antibiotic prescriptions were tested using chi-squared tests for categorical variables and Wilcoxon rank sum tests (Mann-Whitney U test) for continuous variables. A multivariate logistic regression model was conducted to study the association between predefined variables and antibiotic prescription. This model was adjusted for all variables previously listed. Since the regression models contained up to 35 variables, a Bonferroni correction for *p*-value was performed, and a *p* value of <0.001 (calculated as <0.05/35) was considered statistically significant. Furthermore, results were regarded as relevant or not when the odds ratio (OR) was >1.25 or <0.75, respectively. Fitness of the logistic model was checked using the Hosmer–Lemeshow statistic. As a sensitivity analysis, a random effect of practice was estimated.

All analyses were performed separately for patients treated by GPs and by pediatricians (children only). Analyses were carried out using SAS version 9.4 (SAS Institute, Cary, NC, USA).

## 3. Results

### 3.1. Prevalence of Antibiotic Use

**Figure 2:**
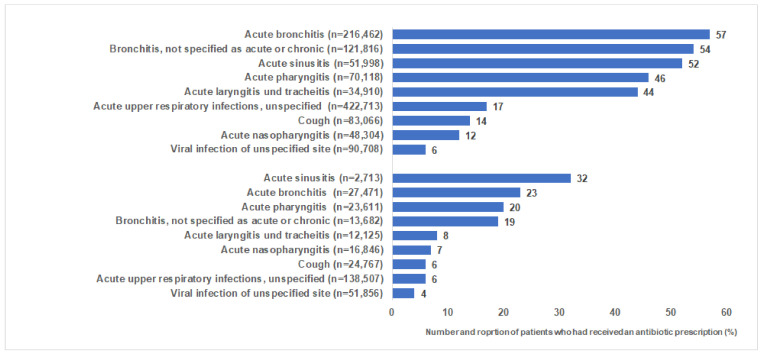
Proportion of patients who had received an antibiotic in general practices (GP) and pediatric practices.

In total, 1,449,154 individuals (1,140,095 in general and 309,059 in pediatrician practices) were diagnosed with ALURTI between January 2015 and March 2019, and 384,410 (26.5%) of them had received an antibiotic prescription on the day of diagnosis. The prevalence of antibiotic prescription was higher in patients treated in general (31.2%) than in pediatric practices (9.1%). By GPs, the highest antibiotic prescription prevalence was observed in patients with acute or non-specified bronchitis (54–57%), acute sinusitis (52%), and acute pharyngitis (46%) (Figure 2). Interestingly, 1.5% of patients with the specific diagnosis of a viral infection of an unspecified site received an antibiotic prescription in GPs, 6.4% in pediatric practices (Figure 2).

When an antibiotic was prescribed, macrolides were the most common drug class by GPs (34%), followed by cephalosporins (27%), and penicillins (21%). In pediatric practices, cephalosporins (44%) had the highest proportion, followed by a penicillin (29%), and a macrolide (26%).

### 3.2. Characteristics of Patients with and without Antibiotic Prescription

When comparing baseline characteristics of patients who had and who had not received an antibiotic prescription in general practices, patients receiving antibiotics were slightly older (51 vs. 46 years) and were more often diagnosed with an acute or non-specified bronchitis, pharyngitis, sinusitis, laryngitis and tracheitis. Additionally, they had more often chronic co-diagnoses, were more often treated in practices preferring antibiotic therapy, and less likely in practices preferring phytopharmaceuticals (Table 1). In pediatric practices, patients receiving antibiotics were also older and more often diagnosed with an acute or non-specified bronchitis, pharyngitis, and sinusitis. Additionally, they had more often asthma diagnoses, were more frequently treated in practices preferring antibiotic therapy, and less frequently treated in practices preferring phytopharmaceuticals (Table 2).

### 3.3. Variables Associated with an Antibiotic Prescription

The results of the multivariate logistic regression model for GPs are displayed in Table 3. The probability of antibiotic prescription increased with higher age, especially for patients aged >60 years (ORs: 1.40–1.56). Diagnoses of acute bronchitis (OR: 6.01), non-specified bronchitis (OR: 5.22), acute sinusitis (OR: 5.32), acute pharyngitis (OR: 4.18), and acute laryngitis and tracheitis (OR: 3.87) were associated with an increased chance of receiving an antibiotic prescription, compared to ALURTI of multiple and unspecified sites. The antibiotic prescription prevalence was lower in Western than in Eastern Germany, while comorbidity obviously had only minor influences. The strongest association with antibiotic prescription was seen in the practice preference for antibiotics (Odds Ratio (OR): 5.23 for >8–10% proportion of antibiotic prescription on all prescriptions per practice per year, and OR: 9.93 for >10% proportion of antibiotic prescription on all prescriptions per practice per year, as compared to a proportion of ≤4%). Conversely, practice preference for phytopharmaceuticals was negatively associated with antibiotic prescription (i.e., OR: 0.44 for >4% of phytopharmaceutical prescription on all prescriptions per practice per year, as compared to ≤1%), as was the diagnosis of a viral infection of unspecified site (OR: 0.32).

In pediatric practices (Table 4), the practice preference for antibiotics was also associated with an antibiotic prescription in patients with ALURTI (OR: 4.00 for >10% proportion of antibiotic prescription on all prescriptions per practice per year, as compared to a proportion of >4–6%). Similar to the situation in general practices, preference for phytopharmaceuticals was negatively associated with antibiotic prescription (OR: 0.20–0.26 for >1% of phytopharmaceutical prescription on all prescriptions per practice per year, as compared to ≤1%), as was the diagnosis of a viral infection of unspecified site (OR: 0.57). Diagnoses of acute sinusitis (OR: 7.14), acute bronchitis (OR: 4.55), acute pharyngitis (OR: 3.57), non-specified bronchitis (OR: 3.43), acute laryngitis and tracheitis (OR: 1.43), acute nasopharyngitis (OR: 1.20), cancer as an underlying disease (OR: 1,81), and practice size of >2000 patients per quarter (OR: 1.35) were positively associated with increased chance of receiving antibiotic prescription. A random effect of practice variable was not significant (OR: 1.00).

## 4. Discussion

This study, conducted in general and pediatric practices in Germany and based on more than one million individuals, resulted in three key findings. First, the recent prevalence of antibiotic prescription in adults with ALURTI was high (31.2%) and associated with age, coded infection diagnosis, and geography. Second, practice-level variables indicating frequent prescription of antibiotics or phytopharmaceuticals had independent associations with antibiotic prescription prevalence, both in general as well as in pediatric practices. Third, antibiotic prescription prevalence among children with ALURTI was low (< 10%) and associated with coded infection diagnosis, cancer as an underlying disease, and practice size in addition to practice preference for antibiotics.

Antibiotics are an important therapy option for bacterial infections and can save lives, provided the correct indication, dose, and duration of therapy. However, in the case of an ALURTI, there is usually no indication for an antibiotic prescription since the majority of such infections are of viral origin, and even in cases of bacterial origin, only a minority of patients benefit from antibiotic treatment. According to a systematic review, there is no difference in the outcomes between patients with immediate and delayed antibiotic use [13]. A more recent randomized trial showed that strategies of no or delayed antibiotic prescription result in similar symptomatic outcomes as immediate prescription and are not associated with patients’ beliefs in the effectiveness of antibiotics [14]. An investigation of patients’ expectations as a reason for prescribing antibiotics for common upper respiratory tract infections reported, that although 11% of patients may have expected an antibiotic prescription, most of them indicated they would trust their physician when he or she deems a prescription unnecessary [15].

Prescribing physician-level factors may be critically affecting antibiotic prescribing for patients with ALURTI. O’Connor et al., for example, identified that a higher number of patients per day resulting in shorter consultations, as well as a doctors’ professional training and their career stage were significant factors [16]. Practice size estimated by the number of patients per practice and quarter as a factor for antibiotic prescribing was also found in other studies from Canada [17] and Norway [18], as well as in the present study, in particular regarding pediatric practices. Other physician-level factors for antibiotic prescribing are diagnostic uncertainty and fear of complications [19]. This may be a reason why the prevalence of antibiotic use in our study was high in acute bronchitis patients, as the likelihood of confusing acute bronchitis and pneumonia is higher than in the case of upper tract respiratory infection [20]. More difficult to understand is the relatively frequent prescription of antibiotics in patients diagnosed with acute sinusitis in the present work. A Cochrane review concluded that the potential benefit of antibiotics to treat acute rhinosinusitis was marginal and that antibiotics are not a first-choice treatment for adults with short-duration sinus infections [21]. In acute non-bacterial laryngitis, the use of antibiotics is also considered not appropriate [22]. We were unable to assess and include clinical severity at presentation as a confounder variable, perhaps explaining the prescription patterns for sinusitis. Coding mild sinusitis as nasopharyngitis (J00) or an acute upper respiratory infection (J06) versus coding more severe or complicated cases as acute sinusitis (J01) may have confounded our analysis. As in other studies [23,24,25], the prevalence of antibiotic prescriptions was higher in individuals with comorbidity, and this may be related to difficulties in distinguishing between bacterial and viral infections in everyday practice and in trying to prevent serious complications [26].

An interesting finding in our study was the association between practice preference for phytotherapy and lower antibiotic prescribing rates, both in adults and children. There is some evidence that the use of phytopharmaceuticals could contribute to a reduction in the number of inappropriate antibiotic prescriptions for respiratory infections; in a recent study based on the electronic medical records from approximately 234,000 primary care and pediatric patients in Germany, the use of selected phytopharmaceuticals in ALURTI was associated with a reduced need for antibiotic prescriptions in the further course of the disease [27]. Reviews of complementary and alternative medicines for treatment of respiratory tract infections indicate that some of the treatments may be effective and safe, and help to reduce unnecessary antibiotic prescribing [28,29,30,31,32,33].

A strength of this study is the large sample size. This is, however, also the study limitation, as every minor difference becomes highly significant with large patient samples. Further limitations include the lack of several potentially confounding factors, such as disease severity, patients’ sociocultural variables, and patients’ preference of antibiotics. An additional limitation of the present study was the unavailability of possible point-of-care test results as a possible confounder. We could not assess the coding accuracy and consistency. The fact that explored covariates are very general, in line with the registry intention of the database, should be noted.

In addition, the study reflects the German healthcare system, and its results may not be generalizable to other countries.

## 5. Conclusions

In conclusion, we show a relatively high recent prevalence of potentially inappropriate antibiotic prescribing for adult patients with ALURTI in a primary care setting. Practice related factors, including antibiotic preference, seemed to play an important role in prescribing prevalence and should be studied in more detail in order to design interventions to further reduce inappropriate antibiotic use.

## Figures and Tables

**Table 1:** Baseline characteristics of study patients in GPs.

Variable	Patients with Antibiotic Prescription (%)	Patients without Antibiotic Prescription (%)	*p* Value
*n*	356,045	784,050	
Age (Mean, SD)	51.2 (20.1)	46.3 (20.7)	<0.001
Age <18	4.5	7.9	<0.001
Age 18–40	26.5	32.4	
Age 41–50	16.1	16.3	
Age 51–60	19.2	18.6	
Age 61–70	14.3	10.6	
Age 71–80	12.3	8.8	
Age >80	7.1	5.4	
Female sex (%)	54.9	54.2	<0.001
Private health insurance coverage (%)	7.2	8.2	<0.001
Acute lower and upper tract respiratory infection diagnosis (%)			
Viral infection of unspecified site	1.5	10.9	<0.001
Acute nasopharyngitis	1.6	5.4	
Acute sinusitis	7.6	3.2	
Acute pharyngitis	9.0	4.9	
Acute laryngitis and tracheitis	4.3	2.5	
Acute upper respiratory infections of multiple and unspecified sites (J06)	19.9	44.9	
Acute bronchitis	34.5	11.9	
Bronchitis, not specified as acute or chronic	18.3	7.2	
Cough	3.3	9.1	
Co-Diagnoses (documented prior to or on the index date) (%)			
Diabetes	6.2	4.4	<0.001
Ischemic heart disease/heart failure	5.3	4.8	<0.001
Renal failure	1.5	1.2	<0.001
Cancer	1.9	1.5	0.352
COPD	3.3	1.9	<0.001
Asthma	3.6	2.7	<0.001
Diagnosis of a bacterial infection within 365–31 days prior to the index date (%)	0.5	0.6	0.098
Number of patients per practice and quarter (%)			
≤1000	18.4	20.7	<0.001
1001–1500	34.4	38.3	
1501–2000	25.4	22.5	
>2000	21.8	18.5	
Western Germany	83.3	82.4	<0.001
Proportion of antibiotic prescription on all prescriptions per practice per year			
≤4%	3.0	8.6	<0.001
>4–≤6%	29.0	45.2	
>6–≤8%	34.8	29.6	
>8–≤10%	19.5	11.9	
>10%	13.7	4.7	
Proportion of phytopharmaceutical prescription on all prescriptions per practice per year (%)			
≤1%	39.4	37.1	<0.001
>1–≤2%	18.7	17.2	
>2–≤3%	15.0	14.9	
>3–≤4%	9.9	10.0	
>4%	17.0	20.8	

**Table 2:** Baseline characteristics of study patients in pediatric practices.

Variable	Patients with Antibiotic Prescription (%)	Patients without Antibiotic Prescription (%)	*p* Value
*n*	28,365	280,694	
Age (Mean, SD)	7.9 (4.7)	7.6 (4.4)	<0.001
Age <18			<0.001
Age 2–5	40.2	39.1	
Age 6–12	38.7	44.5	
Age 13–17	21.1	16.4	
Female sex (%)	49.4	48.4	1.000
Private health insurance coverage (%)	9.6	9.5	0.635
Acute lower and upper tract respiratory infection diagnosis (%)			
Viral infection of unspecified site	6.4	17.7	<0.001
Acute nasopharyngitis	4.2	5.6	<0.001
Acute sinusitis	3.1	0.6	<0.001
Acute pharyngitis	16.3	6.7	<0.001
Acute laryngitis and tracheitis	3.5	3.9	<0.001
Acute upper respiratory infections of multiple and unspecified sites (J06)	29.8	46.0	<0.001
Acute bronchitis	22.2	7.5	<0.001
Bronchitis, not specified as acute or chronic	9.3	3.9	<0.001
Cough	5.2	8.1	<0.001
Co-Diagnoses (documented prior to or on the index date) (%)			
Diabetes	0.1	0.1	0.294
Ischemic heart disease/heart failure	0.1	0.1	0.096
Renal failure	0.0	0.0	0.723
Cancer	0.1	0.1	0.680
COPD	4.7	4.2	<0.001
Asthma	3.4	2.9	<0.001
Diagnosis of a bacterial infection within 365–31 days prior to the index date (%)	7.4	6.5	<0.001
Number of patients per practice and quarter (%)			
≤1000	9.0	10.1	<0.001
1001–1500	34.5	37.5	
1501–2000	34.9	35.6	
>2000	21.6	16.8	
Western Germany (%)	85.6	83.0	<0.001
Proportion of antibiotic prescription on all prescriptions per practice per year (%)			
≤4%	0.0	1.5	<0.001
>4–≤6%	4.5	11.4	
>6–≤8%	14.9	23.3	
>8–≤10%	27.8	29.0	
>10%	52.9	34.7	
Proportion of phytopharmaceutical prescription on all prescriptions per practice per year (%)			
≤1%	22.5	21.0	<0.001
>1–≤10	32.9	31.6	
>10–≤15%	23.8	24.2	
>15%	20.8	23.2	

**Table 3:** Variables associated with an antibiotic prescription in patients diagnosed with an acute lower or upper respiratory tract infection in GPs.

Variable	Odds Ratio (95% CI)	*p* Value *
Age 18–40	Reference	
Age 41–50	1.20 (1.18–1.22)	<0.001
Age 51–60	1.25 (1.23–1.27)	<0.001
Age 61–70	1.55 (1.52–1.58)	<0.001
Age 71–80	1.56 (1.53–1.59)	<0.001
Age >80	1.40 (1.37–1.44)	<0.001
Female	Reference	
Male	0.99 (0.98–1.00)	0.088
Statutory health insurance	Reference	
Private health insurance	0.89 (0.87–0.91)	<0.001
Acute upper respiratory infections of multiple and unspecified sites	Reference	
Viral infection of unspecified site	0.32 (0.32–0.33)	<0.001
Acute nasopharyngitis	0.66 (0.64–0.68)	<0.001
Acute sinusitis	5.32 (5.21–5.44)	<0.001
Acute pharyngitis	4.18 (4.10–4.27)	<0.001
Acute laryngitis and tracheitis	3.87 (3.77–3.98)	<0.001
Acute bronchitis	6.01 (5.93–6.10)	<0.001
Bronchitis, not specified as acute or chronic	5.22 (5.13–5.30)	<0.001
Cough	0.77 (0.75–0.79)	<0.001
Co-Diagnoses (documented prior to or on the index date)		
Diabetes	1.07 (1.05–1.09)	<0.001
Ischemic heart disease/heart failure	1.01 (0.98–1.03)	0.492
Renal failure	0.90 (0.86–0.93)	<0.001
Cancer	0.97 (0.93–1.00)	0.061
COPD	1.24 (1.20–1.27)	<0.001
Asthma	1.13 (1.10–1.16)	<0.001
Diagnosis of a bacterial infection within 365–30 days prior to the index date	1.00 (0.93–1.07)	0.894
Number of patients per practice and quarter		
≤1000	Reference	
1001–1500	1.03 (1.02–1.05)	<0.001
1501–2000	1.03 (1.01–1.04)	<0.001
>2000	1.08 (1.06–1.10)	<0.001
Western Germany	0.73 (0.72–0.74)	<0.001
Eastern Germany	Reference	
Proportion of antibiotic prescription on all prescriptions per practice per year		
≤4	Reference	
>4–≤6	2.16 (2.10–2.21)	<0.001
>6–≤8	3.95 (3.84–4.05)	<0.001
>8–≤10	5.23 (5.09–5.38)	<0.001
>10	9.93 (9.62–10.24)	<0.001
Proportion of phytopharmaceutical prescription on all prescriptions per practice per year		
≤1	Reference	
>1–≤2	0.89 (0.88–0.90)	<0.001
>2–≤3	0.64 (0.63–0.66)	<0.001
>3–≤4	0.66 (0.64–0.68)	<0.001
>4	0.44 (0.43–0.46)	<0.001

* Significance values <0.001 were recorded when the odds ratio (OR) was >1.25 or <0.75, respectively, as these effects were regarded as relevant.

**Table 4:** Variables associated with an antibiotic prescription in patients diagnosed with an acute lower and upper respiratory tract infection diagnosis in pediatric practices.

Variable	Odds Ratio (95% CI)	*p* Value *
Age 2–5	Reference	
Age 6–12	0.85 (0.82–0.88)	<0.001
Age 13–17	1.21 (1.16–1.26)	<0.001
Female sex	Reference	
Male sex	0.94 (0.91–0.97)	<0.001
Statutory health insurance coverage	Reference	
Private health insurance coverage	1.03 (0.98–1.09)	0.289
Acute lower and upper respiratory tract infection diagnosis		
Viral infection of unspecified site	0.57 (0.54–0.61)	<0.001
Acute nasopharyngitis	1.20 (1.12–1.29)	<0.001
Acute sinusitis	7.14 (6.44–7.91)	<0.001
Acute pharyngitis	3.57 (3.39–3.75)	<0.001
Acute laryngitis and tracheitis	1.43 (1.32–1.55)	<0.001
Acute upper respiratory infections of multiple and unspecified sites (J06)	Reference	
Acute bronchitis	4.55 (4.35–4.76)	<0.001
Bronchitis, not specified as acute or chronic	3.43 (3.23–3.64)	<0.001
Cough	1.18 (1.11–1.26)	<0.001
Co-Diagnoses (documented prior to or on the index date)		
Diabetes	0.89 (0.59–1.32)	0.552
Ischemic heart disease/heart failure	1.75 (1.10–2.78)	0.033
Renal failure	1.07 (0.24–4.83)	0.926
Cancer	1.81 (1.15–2.86)	0.019
COPD	1.10 (1.03–1.18)	0.005
Asthma	0.88 (0.82–0.95)	0.002
Diagnosis of a bacterial infection within 365–30 days prior to the index date	1.09 (1.03–1.16)	0.002
Number of patients per practice and quarter		
≤1000	Reference	
1001–1500	1.14 (1.07–1.21)	<0.001
1501–2000	1.20 (1.13–1.27)	<0.001
>2000	1.35 (1.26–1.43)	<0.001
Eastern Germany	Reference	
Western Germany	1.07 (1.02–1.13)	0.006
Proportion of antibiotic prescription on all prescriptions per practice per year		
>4–≤6	Reference	
>6–≤8	1.62 (1.49–1.75)	<0.001
>8–≤10	2.41 (2.24–2.60)	<0.001
>10	4.00 (3.71–3.30)	<0.001
Proportion of phytopharmaceutical prescription on all prescriptions per practice per year		
≤1	Reference	
>1–≤10	0.20 (0.18–0.23)	<0.001
>10–≤15	0.24 (0.87–0.97)	<0.001
>15	0.26 (0.23–0.30)	<0.001

* Significance values <0.001 were recorded when the odds ratio (OR) was >1.25 or <0.75, respectively, as these effects were regarded as relevant.

## Data Availability

Data is contained within the article.
